# Undenatured type II collagen (UC-II®) for joint support: a randomized, double-blind, placebo-controlled study in healthy volunteers

**DOI:** 10.1186/1550-2783-10-48

**Published:** 2013-10-24

**Authors:** James P Lugo, Zainulabedin M Saiyed, Francis C Lau, Jhanna Pamela L Molina, Michael N Pakdaman, Arya Nick Shamie, Jay K Udani

**Affiliations:** 1 InterHealth Nutraceuticals, Benicia, CA 94510, USA; 2 Medicus Research LLC, 28720 Roadside Drive, Suite 310, Agoura Hills, CA 91301, USA; 3 UCLA Medical Center, Santa Monica, CA 90401, USA; 4 Northridge Hospital Integrative Medicine Program, Northridge, CA 91325, USA

**Keywords:** UC-II, Undenatured type II collagen, Joint function, Knee extension, Stepmill, Joint pain

## Abstract

**Background:**

UC-II contains a patented form of undenatured type II collagen derived from chicken sternum. Previous preclinical and clinical studies support the safety and efficacy of UC-II in modulating joint discomfort in osteoarthritis and rheumatoid arthritis. The purpose of this study was to assess the efficacy and tolerability of UC-II in moderating joint function and joint pain due to strenuous exercise in healthy subjects.

**Methods:**

This randomized, double-blind, placebo-controlled study was conducted in healthy subjects who had no prior history of arthritic disease or joint pain at rest but experienced joint discomfort with physical activity. Fifty-five subjects who reported knee pain after participating in a standardized stepmill performance test were randomized to receive placebo (n = 28) or the UC-II (40 mg daily, n = 27) product for 120 days. Joint function was assessed by changes in degree of knee flexion and knee extension as well as measuring the time to experiencing and recovering from joint pain following strenuous stepmill exertion.

**Results:**

After 120 days of supplementation, subjects in the UC-II group exhibited a statistically significant improvement in average knee extension compared to placebo (81.0 ± 1.3º vs 74.0 ± 2.2º; p = 0.011) and to baseline (81.0 ± 1.3º vs 73.2 ± 1.9º; p = 0.002). The UC-II cohort also demonstrated a statistically significant change in average knee extension at day 90 (78.8 ± 1.9º vs 73.2 ± 1.9º; p = 0.045) versus baseline. No significant change in knee extension was observed in the placebo group at any time. It was also noted that the UC-II group exercised longer before experiencing any initial joint discomfort at day 120 (2.8 ± 0.5 min, p = 0.019), compared to baseline (1.4 ± 0.2 min). By contrast, no significant changes were seen in the placebo group. No product related adverse events were observed during the study. At study conclusion, five individuals in the UC-II cohort reported no pain during or after the stepmill protocol (p = 0.031, within visit) as compared to one subject in the placebo group.

**Conclusions:**

Daily supplementation with 40 mg of UC-II was well tolerated and led to improved knee joint extension in healthy subjects. UC-II also demonstrated the potential to lengthen the period of pain free strenuous exertion and alleviate the joint pain that occasionally arises from such activities.

## Introduction

The impact of strenuous exercise on knee joints may cause localized pain and stiffness, which are hallmark features of pathologic inflammatory disease [1]. It has been shown that when dogs undergo a strenuous running regimen, significant losses in articular cartilage and glycosaminoglycans occur [2]. Such studies suggest that strenuous exercise may activate some of the same physiological processes that occur in arthritic disease [2-4]. In fact, *in vitro* studies have shown that many of the cytokines implicated in the onset and progression of both rheumatoid arthritis (RA) and osteoarthritis (OA) also appear to regulate the remodeling of the normal knee extracellular matrix (ECM) following strenuous exertion [5].

When normal chondrocytes undergo strenuous mechanical stimulation under static conditions, their physiology shifts towards ECM breakdown, as indicated by the upregulation of several metalloproteinases (MMPs), such as MMP-13 as well as tumor necrosis factor (TNF)-α, interleukin (IL)-1β, IL-6, and various aggrecanases [5,6]. This *in vitro* catabolic response is mediated by changes in the phosphorylation, expression, or translocation of several transcription factors to the cell nucleus such as NF-κB, p38 MAPK, Akt, and ERK [7,8]. By contrast, normal chondrocytes produce the anti-inflammatory cytokine IL-4 when mechanically stimulated under moderate and dynamic conditions [9]. The secretion of this autocrine molecule not only helps in shifting chondrocyte metabolism towards the synthesis of aggrecan and type II collagen, it also downregulates production of nitric oxide (NO) and various MMPs and aggrecanases [10-12]. This conclusion is corroborated by the finding that pretreatment of strenuously compressed normal chondrocytes with IL-4 attenuates their catabolic response [11]. This suggests that IL-4 plays a key role in downregulating remodeling functions, restoring articular cartilage homeostasis, as well as decreasing chondrocyte apoptosis following strenuous mechanical loading [12,13].

Mechanically stressed chondrocytes also produce a number of other molecules known to participate in inflammatory responses, including prostoglandin E2, NO, and vascular endothelial growth factor [14]. These are proinflammatory molecules that, in conjunction with TNF-α, IL-6 and IL-1β, result in a localized, and transitory inflammatory-like response that is part of the normal repair process occurring in knee joints, serves to moderate remodeling events [3]. Ostrowski et al. [15] showed that healthy individuals express up to 27-fold greater concentrations of the anti-inflammatory cytokine IL-10 in blood following a marathon run when compared to IL-10 blood levels at rest. This finding is not surprising given that these same individuals also show marked increases in the proinflammatory cytokines TNF-α, IL-1β, and IL-6. It therefore appears that in healthy subjects undergoing strenuous exertion, the induction of proinflammatory cytokines is offset by the synthesis of anti-inflammatory agents as part of the recovery process. This view is supported by the observation that IL-10 reduces the catabolic impact of IL-1β and TNF-α on cartilage explants from healthy volunteers, and this effect is enhanced by combining IL-10 with IL-4 [13].

Another protein released by dynamically compressed chondrocytes is transforming growth factor (TGF)-β [16-18]. This factor is secreted by many cell types and is known to interfere with the cell cycle and arrest differentiation [19]. With regard to chondrocytes, TGF-β induces cell proliferation *in vitro* and slows terminal differentiation into hypertrophic cells [20]. Numerous studies have shown that TGF-β reverses the *in vitro* catabolic effect of various proinflammatory cytokines on normal chondrocytes as well as chondrocytes harvested from RA and OA donors [21-23].

The overall findings discussed above point to a new, unifying view of joint physiology. It suggests that many of the biological processes occurring in knee joints affected by RA and OA also participate in the maintenance of healthy knees [1,4,5]. It therefore seems appropriate to test the efficacy of natural supplements or ingredients, which have been shown to moderate joint pain in RA and OA, as possible candidates for treating the joint discomfort that occasionally results from strenuous exercise in healthy individuals.

UC-II is a natural ingredient which contains a glycosylated, undenatured type-II collagen [24]. Previous studies have shown that small doses of UC-II modulate joint health in both OA and RA [24-26]. Tong et al. [27], using an *in vivo* model of collagen induced arthritis (CIA), demonstrated that ingesting microgram quantities of undenatured type II collagen significantly reduces circulating levels of inflammatory cytokines, potentially serving to decrease both the incidence and the severity of arthritis [28]. The ability to alter immunity via the ingestion of a food, or an antigen, is called oral tolerance. This is an ongoing normal physiological process that protects the alimentary tract against untoward immunological damage [29,30]. Research into its mechanism of action has revealed that several distinct types of T regulator cells mediate this phenomenon by releasing IL-10 and TGF-β [30]. It has also been shown that this effect is transitory in nature requiring that the food, or antigen, be consumed continuously in order to maintain the tolerogenic state [30]. Given these findings, plus our current understanding of the role of various cytokines in normal joint physiology, it was hypothesized that supplementation with UC-II might relieve joint discomfort and restore joint function in healthy subjects.

The aim of this randomized, double blind, placebo-controlled study was to assess the impact of UC-II on knee function in otherwise healthy subjects with no prior history of arthritic disease who experienced knee pain upon strenuous physical exertion. The primary efficacy variable for assessing knee function included measurements of flexibility using range of motion (ROM) goniometry.

## Methods

### Investigational product

The investigational study product UC-II is derived from chicken sternum. It is manufactured using a patented, low-temperature process to preserve its native structure. For the clinical study, 40 mg of UC-II material (Lot 1109006), which provides 10.4 ± 1.3 mg of native type-II collagen, was encapsulated in an opaque capsule with excipients. Placebo was dispensed in an identical capsule containing only excipients (microcrystalline cellulose, magnesium stearate and silicon dioxide). Both study materials were prepared in a good manufacturing practice (GMP)-certified facility and provided by InterHealth Nutraceuticals, Inc. (Benicia, CA). Subjects were instructed to take one capsule daily with water before bedtime.

### Recruitment of subjects

One hundred and six subjects were screened for eligibility using the inclusion–exclusion criteria defined in Table 1. Only healthy adults who presented with no knee joint pain at rest and no diagnosable markers indicative of active arthritic disease, as outlined by the American College of Rheumatology (ACR) guidelines [31,32], were admitted into the study. To accomplish this, all potential subjects were screened by a board certified clinician. Subjects presenting with any knee pain at rest and at least 3 of 6 clinical classification criteria, which included age greater than 50 years, morning stiffness in the joint lasting 30 minutes or less, crepitus on knee joint manipulation, body tenderness, bony enlargements, knee swelling or presence of excess fluid, and palpable warmth, were excluded. Potential subjects reporting the occasional use of NSAIDs, other pain relief medication, or anti-inflammatory supplements underwent a 2-week washout period before randomization.

**Table 1:** Inclusion–exclusion criteria


**Inclusion**	
•	Subject must be ≥30 and ≤65 years of age
•	Body mass index (BMI) must be ≥18 and ≤35 kg/m^2^
•	Knee joint criteria: (1) no knee joint discomfort at rest; (2) must achieve a knee joint discomfort score of at least 5 on an 11-point Likert scale within 10 minutes of initiating the stepmill protocol
•	Maintain existing food and physical activity patterns throughout the study period
•	Judged by Investigator to be in general good health on the basis of medical history
•	Subject understands the study procedures and provides signed informed consent to participate in the study and authorizes the release of relevant health information to the study investigator
•	Females of child bearing age must agree to use approved birth control methods during the study
**Exclusion**	
•	Subjects with any indicators of arthritis, joint disorders, or history of immune system or autoimmune disorders
•	Daily use of NSAIDs; however, daily use of 81 mg of aspirin for cardioprotection is allowed
•	Daily use of anti-inflammatory or omega-3-fatty acid dietary supplements or using supplements to maintain joint health 30 days prior to screening
•	Subjects with a history of knee or hip joint replacement surgery, or any hip or back pain which interferes with ambulation
•	Use of any immunosuppressive drugs in the last 12 months (including steroids or biologics)
•	Glucocorticoid injection or hyaluronic acid injection in affected knee within 3 months prior to enrollment
•	History of surgery or significant injury to the target joint within 6 months prior to study enrollment, or an anticipated need for surgical or invasive procedure that will be performed during the study
•	Subjects with a chronic pain syndrome and in the judgment of the Investigator is unlikely to respond to any therapy
•	Participation in a clinical study with exposure to any non-registered drug product within 30 days prior
•	Subjects who have any physical disability which could interfere with their ability to perform the functional performance measures included in this protocol
•	Any significant GI condition that would potentially interfere with the evaluation of the study product
•	Clinically significant renal, hepatic, endocrine (including diabetes mellitus), cardiac, pulmonary, pancreatic, neurologic, hematologic, or biliary disorder
•	Subjects with vascular condition which interferes with ambulation
•	Known allergy or sensitivity to herbal products, soy or eggs
•	Vegetarian or Vegan
•	History or presence of cancer in the prior two years, except for non-melanoma skin cancer.
•	Individual has a condition the Investigator believes would interfere with his or her ability to provide informed consent, comply with the study protocol, which might confound the interpretation of the study results or put the person at undue risk
•	Untreated or unstable hypothyroidism, an active eating disorder, or evidence of any neurological disorders
•	Recent history of (within 12 months) or strong potential for alcohol or substance abuse
•	Females who are pregnant, lactating, or unwilling to use adequate contraception during the study

Subjects were required to undergo a 10 minute period of performance testing using a standardized stepmill test developed and validated by Medicus Research (Udani JK, unpublished observation). It involved exercising at level 4 on a StepMill® model 7000PT (StairMaster® Health & Fitness Products, Inc., Kirkland, WA) until one or both knees achieved a discomfort level of 5 on an 11 point (0–10) Likert scale [33]. This pain threshold had to be achieved within a 10 minute period otherwise the subject was excluded. Once the requisite pain level was achieved the subject was asked to continue stepping for an additional two minutes in order to record the maximum pain level achieved before disembarking from the stepmill. The following knee discomfort measures were recorded from the start of the stepmill test: (1) time to onset of initial joint pain; (2) time to onset of maximum joint pain; (3) time to initial improvement in knee joint pain; (4) time to complete recovery from knee joint pain. Subjects who experienced a pain score of 5 (or greater) within one minute of starting the stress test were excluded. Out of 106 screened candidates, 55 subjects were enrolled in the study. Each subject voluntarily signed the IRB-approved informed consent form. After enrollment, the subjects were randomly assigned to either the placebo or the UC-II group.

### Study design and trial site

This randomized, double blind, placebo-controlled study was conducted at the Staywell Research clinical site located in Northridge, CA. Medicus Research (Agoura Hills, CA) was the contract research organization (CRO) of record. The study protocol was approved by Copernicus Group IRB (Cary, NC) on April 25th 2012. The study followed the principles outlined in the Declaration of Helsinki (version 1996).

### Randomization and blinding

Simple randomization was employed using a software algorithm based on the atmospheric noise method (http://www.random.org). Sequential assignment was used to determine group allocation. Once allocated, the assignment was documented and placed in individually numbered envelopes to maintain blinding. Subjects, clinical staff, plus data analysis and management staff remained blinded throughout the study.

### Study schedule

**Figure 1:**
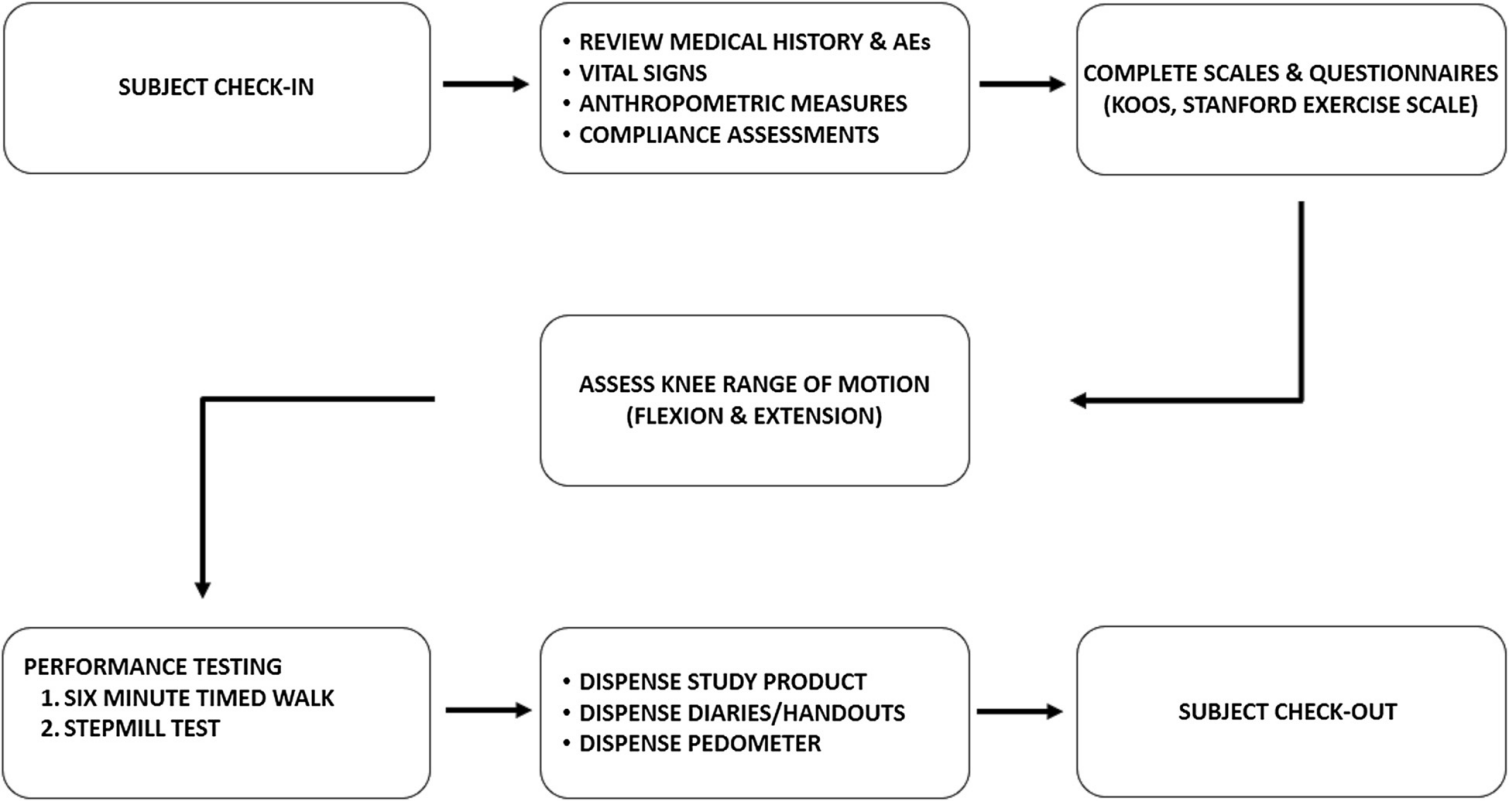
Sequence of study procedures at a typical clinic visit.

The study duration was 17 weeks with a total of 7 visits that included screening, baseline, days 7, 30, 60, 90 and 120 (final visit). Table 2 summarizes the study visits and activities. Figure 1 depicts the sequence of study procedures that subjects underwent during each visit. All subjects completed a medical history questionnaire at baseline and compliance reports during follow-up evaluations at 7, 30, 60, 90 and 120 days. Subjects were assessed for anthropometric measures, vital signs, knee range of motion (flexion and extension), six-minute timed walk, as well as the onset and recovery from pain using the Udani Stepmill Procedure. A Fitbit (San Francisco, CA) device was used to measure daily distance walked, steps taken and an average step length for study participants. Subjects were also asked to complete the KOOS survey as well as the Stanford exercise scales.

**Table 2:** Protocol summary

**Protocol activities**	**V1**	**V2**	**V3**	**V4**	**V5**	**V6**	**V7**
	**Day −7**	**Day 0**	**Day 7**	**Day 30**	**Day 60**	**Day 90**	**Day 120**
	**Screen**	**Baseline**					**End**
Informed consent	x						
Inclusion/Exclusion	x						
Medical history and physical exam	x						
Vital signs/anthropometric measures	x	x	x	x	x	x	x
Urine pregnancy test	x	x					
Administer and review scales/questionnaires/diaries	x	x	x	x	x	x	x
Stressor (Udani Stepmill protocol)	x	x	x	x	x	x	x
Functional measures (6-min timed walk)	x	x	x	x	x	x	x
Goniometry (range of motion)		x	x	x	x	x	x
Review concomitant therapies	x	x	x	x	x	x	x
Intercurrent medical issues review		x	x	x	x	x	x
Compliance assessment (including phone calls)		x	x	x	x	x	x
Randomization		x					
Study supplement preparation & dispensing		x	x	x	x	x	

### Knee range of motion measurements

Knee extension was measured by goniometry. Briefly, subjects were instructed to sit in an upright position on a table edge with their backs straight (knee position defined as 90°). The axis of a goniometer was placed at the intersection of the thigh and shank at the knee joint. Subjects were asked to bring their knees to full extension without changing the position of the pelvis and lumbar spine. The extended knee joint angle was measured and recorded. For knee flexion measurement, subjects were asked to actively flex their knees while lying in a prone position with their shins off the end of the table. The range of knee flexion motion was then measured and documented.

### Timed joint discomfort measurements

Briefly, a stopwatch was started when subjects began climbing the stepmill. Time to onset of pain was recorded at the first sign of pain in the target knee. The baselines at each time point were normalized to account for dropouts. Percent change in time to complete recovery from pain was measured as follows: a new stopwatch was started when the subjects disembarked from the stepmill and the time to complete recovery from pain was recorded. The baselines at each time point were normalized to account for dropouts then compared against the reference interval which was defined as the percentage change between the study baseline and day 7.

### KOOS knee survey & Stanford exercise scales

The KOOS survey is a validated instrument consisting of 42 questions that are classified into sub-scales such as symptoms, stiffness, pain, daily activities, recreational activities and quality of life [34]. It measures the subjects’ opinion about their knees and their ability to perform daily activities during the past week. The Stanford exercise behavior scale comprises 6 questions designed to assess exercise behaviors during the previous week [35].

### Six minute timed walk

Subjects were instructed to walk up and down a hallway for 6 minutes as rapidly as possible without causing any pain. A measuring wheel (RoadRunner Wheel, Keson Industries, Aurora, IL) was used to measure distance travelled in 6 minutes.

### Rescue medication

No rescue medications were allowed during the course of the study. At all study visits, subjects were given a list of the 43 prohibited medications and supplements (Table 3). Changes in overall medication history, or the use of these substances, were then recorded by the study coordinator. Subjects found to have used any of these prohibited substances were excluded from further participation in the study as per protocol.

**Table 3:** Representative list of prohibited medications* by category

**Category**	**Medications**
Joint supplements (Omega-3, Omega-6 plus others)	Alpha-Linolenic acid
	Docosapentaenoic acid
	Docosahexaenoic acid
	Eicosatrienoic acid
	Eicosatetraenoic acid
	Eicosapentaenoic acid
	Hexadecatrienoic acid
	Heneicosapentaenoic acid
	Stearidonic acid
	Tetracosapentaenoic acid
	Tetracosahexaenoic acid
	Glucosamine (all forms)
	Chondroitin (all forms)
	Other herbal ingredients
NSAIDs (OTC and prescription)	Aspirin
	Diflunisal
	Diclofenac
	Celecoxib
	Etodolac
	Fenoprofen
	Flurbiprofen
	Ibuprofen
	Indomethacin
	Ketoprofen
	Meclofenamate
	Mefenamic acid
	Meloxicam
	Nabumetone
	Naproxen
	Oxaprozin
	Piroxicam
	Rofecoxib
	Sulindac
	Tolmetin
	Valdecoxib

*Selected from a list of 43 prohibited medications and supplements.

### Statistics

Outcome variables were assessed for conformance to the normal distribution and transformed as required. Within group significance was analyzed by non-parametric Sign test or by non-parametric Wilcoxon Signed Rank test, while Wilcoxon Mann–Whitney test was used to analyze between groups significance. The Fisher Exact test was used to evaluate the complete loss of pain between study cohorts whereas the binomial test was used to assess the likelihood of complete loss of pain at each visit. P-values equal to or less than 0.05 were considered statistically significant. All analyses were done on a per protocol basis using SPSS, v19 (IBM, Armonk, NY). Results were presented as mean ± SEM.

## Results

### Baseline demographics

A total of 55 individuals met the eligibility criteria and were randomized to the placebo (n = 28) or to the UC-II (n = 27) group. Baseline demographic characteristics for subjects in both groups were similar with respect to age, gender, height, weight and BMI (Table 4). A total of nine subjects, three in UC-II group and six in placebo group, were lost to follow-up. The results presented herein encompass 46 total subjects, 22 subjects in the placebo group plus 24 subjects in the UC-II group. It should be noted that the average age of the study participants was approximately 46 years which is about 16 years younger than the average age observed in many OA studies [36-38].

**Table 4:** Demographic and baseline characteristics of enrolled subjects

**Characteristics**	**UC-II**	**Placebo**
Total number of subjects	27	28
Number of males	11	12
Number of females	16	16
Age (years)	46.1 ± 1.5	46.6 ± 1.8
Weight (kg)	75.5 ± 2.9	77.5 ± 3.1
Height (cm)	167.1 ± 2.0	168.4 ± 2.0
BMI (kg/m^2^)	26.8 ± 0.8	27.1 ± 0.7

Values are expressed as Mean ± SEM.

### Knee extension and flexion

**Figure 2:**
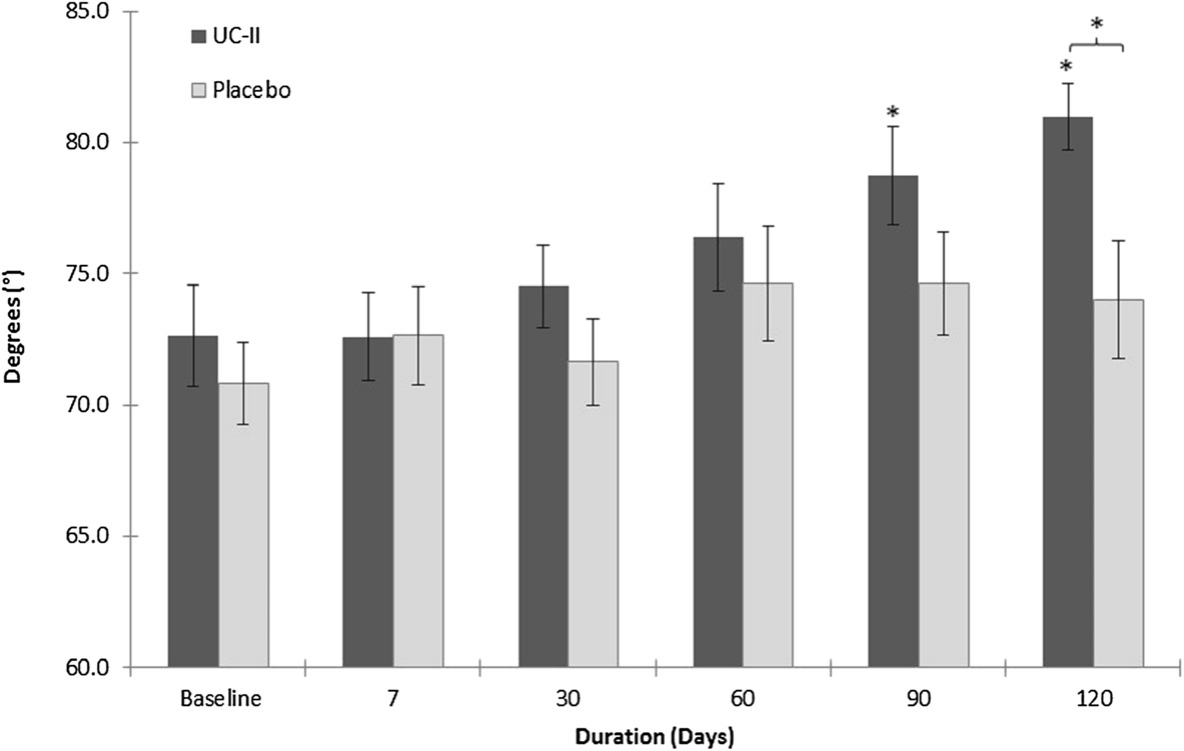
**Knee extension as measured by goniometry.** Values are presented as Mean ± SEM. *p ≤ 0.05 indicates a statistically significant difference versus baseline or placebo. Number of completers: n = 24 in UC-II group (n = 3 dropouts); n = 20 in placebo group (n = 6 dropouts; n = 2 did not participate in ROM assessment).

Figure 2 summarizes the average knee extension changes over time for subjects supplemented with either UC-II or placebo. The UC-II supplemented cohort presented with a statistically significant greater increase in the ability to extend the knee at day 120 as compared to the placebo group (81.0 ± 1.3º vs 74.0 ± 2.2º, p = 0.011) and to baseline (81.0 ± 1.3º vs 73.2 ± 1.9º, p = 0.002). The UC-II group also demonstrated a significant increase in knee extension at day 90 (78.8 ± 1.9º vs 73.2 ± 1.9º, p = 0.045) compared to baseline only. An intent to treat (ITT) analysis of these data also demonstrated a statistically significant net increase in knee extension at day 120 versus placebo (80.0 ± 1.3º vs 73.7 ± 1.8º, p = 0.006). No statistically significant changes were observed in the placebo group at any time during this study. With respect to knee flexion, no significant changes were noted in either study group (p > 0.05). The power associated with the former per protocol statistical analyses was 80%.

### Time to onset of initial joint pain

**Figure 3:**
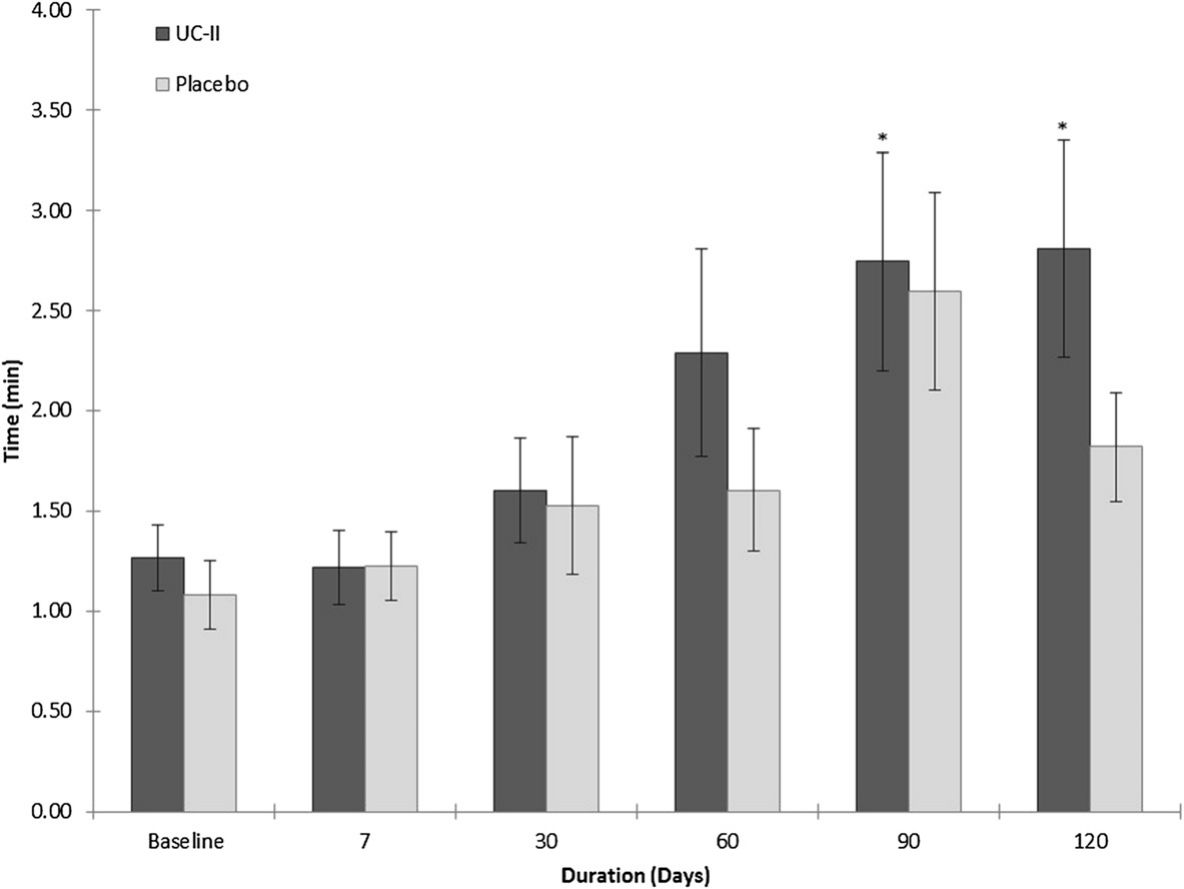
**Impact of stepmill procedure on the onset of pain.** Values are presented as Mean ± SEM. *p ≤ 0.05 indicates a statistically significant difference from baseline. Number of completers: n = 19 in UC-II group (n = 3 dropouts; n = 5 did not have pain); n = 20 in placebo group (n = 6 dropouts; n = 1 did not have pain; n = 1 did not use stepmill).

Supplementation with UC-II resulted in statistically significant increases in the time to onset of initial joint pain at day 90 (2.75 ± 0.5 min, p = 0.041) and at day 120 (2.8 ± 0.5 min, p = 0.019) versus a baseline of 1.4 min for each visit. No statistically significant differences were noted for either the placebo group or between groups (Figure 3).

Five individuals in the UC-II group and one in the placebo group reported no onset of pain by the end of study (see below and Table 5). Given this unexpected finding, an additional analysis was undertaken which included these individuals in the time to onset of initial pain analysis. The 10 minute limit of the stepmill procedure was used as the lower limit to pain onset. Under these conservative assumptions, supplementation with UC-II yielded statistically significant increases in time to onset of pain at day 90 (3.65 ± 0.7 min, p = 0.011) and day 120 (4.31 ± 0.7 min, p = 0.002) versus a baseline of 1.4 min for each visit. The between-group comparison at day 120 approached the statistical level of significance favoring the UC-II cohort (p = 0.051).

**Table 5:** Subjects reporting complete loss of knee pain on stepmill test

**Visit**	**UC-II**			**Placebo**		
	**No. of pain free subjects (%)**	**Continuity of pain loss**^**#**^	**P value (Binomial test)**	**No. of pain free subjects (%)**	**Continuity of pain loss**^**#**^	**P value (Binomial test)**
Baseline	0.0 (0)	0	NA	0.0 (0)	0	NA
Day 7	0.0 (0)	0	NA	0.0 (0)	0	NA
Day 30	1.0 (4)	1N	0.5	0.0 (0)	0	NA
Day 60	3.0 (13)	1R, 2N	0.125	0.0 (0)	0	NA
Day 90	3.0 (13)	2R, 1N	0.125	1 (5)	1N	0.5
Day 120	5.0 (21)	3R, 2N	0.031^†^	1 (5)	1R	0.5

Values denote number of subjects while parenthesis provides the percent of total subjects who did not have any pain on stepmill. Continuity indicates the number of subjects in whom the absence of pain was maintained across visits. ^†^Significant at p ≤ 0.05 based on independent binomial testing of each visit using the null hypothesis that the probability of a subject experiencing no joint pain is equal to zero. There was no statistical difference between groups. ^*#*^*R* Repeat subject (i.e. same subject who reported no pain in previous visit), *N* New subject who reports no pain for the first time.

### Time to onset of maximum joint pain

A statistically significant difference between groups was noted at day 60 (6.39 ± 0.5 min vs 4.78 ± 0.5 min; p = 0.025) favoring the UC-II cohort. This significance did not persist during the remainder of the study suggesting that this was a random occurrence.

### Time to initial improvement in knee joint pain

The time to offset of joint pain was recorded immediately upon the subject stepping off the stepmill. Both groups began to recover from pain with the same rate resulting in no significant differences between groups in the time to initial offset of joint pain (p > 0.05).

### Time to complete recovery from knee joint pain

**Figure 4:**
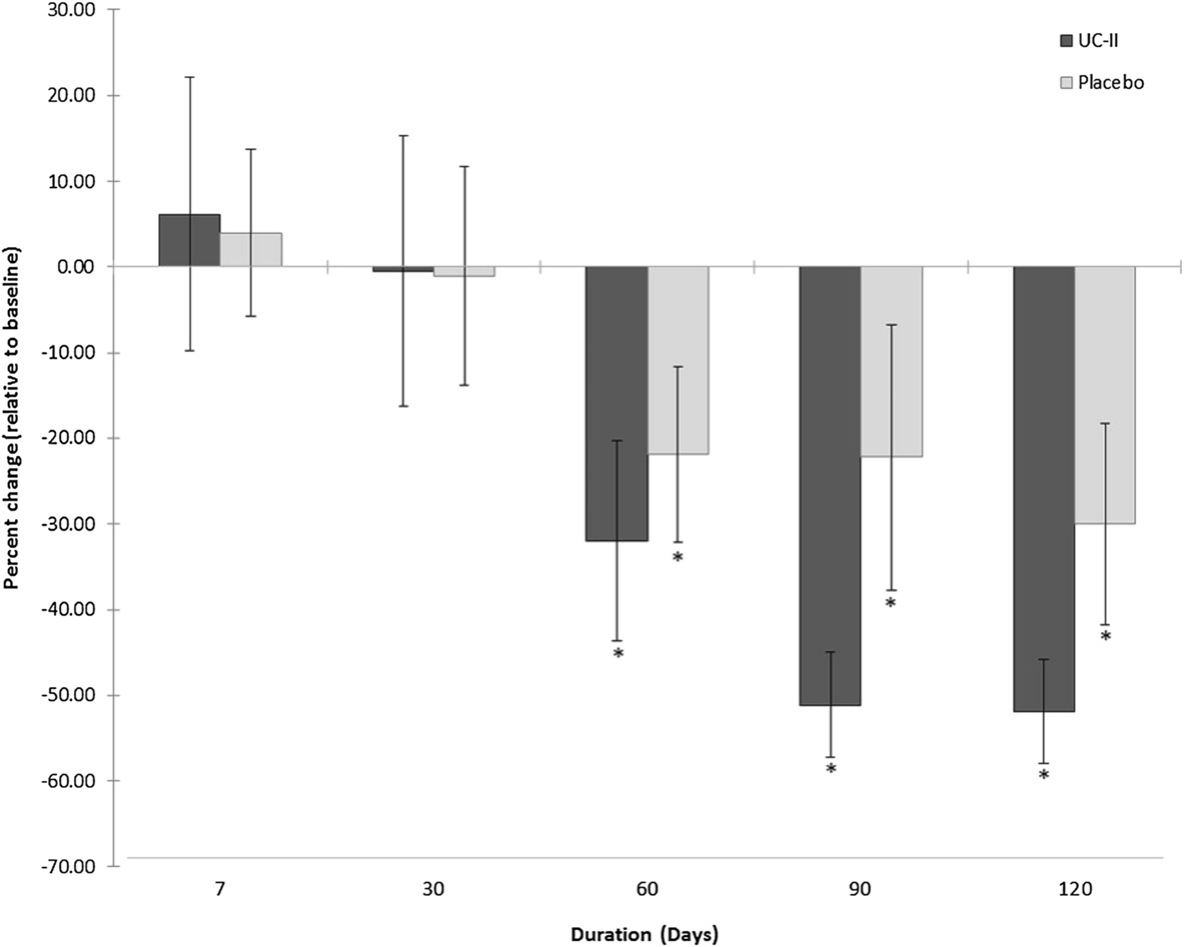
**Percent change in time to complete recovery from pain.** Values are presented as Mean ± SEM. *p ≤ 0.05 indicates a statistically significant difference from baseline. Number of completers: n = 18 in UC-II group (n = 3 dropouts; n = 5 did not have pain; n = 1 time to complete recovery from pain was not achieved); n = 20 in placebo group (n = 6 dropouts; n = 1 did not have pain; n = 1 did not use stepmill).

The time to complete recovery from joint pain showed significant reductions at days 60, 90 and 120 compared to baseline for both the UC-II group as well as the placebo group (Figure 4). Percent changes in times were calculated after normalizing the baselines against the reference range of baseline to day 7. The UC-II group exhibited average reductions of 31.9 ± 11.7% (p = 0.041), 51.1 ± 6.1% (p = 0.004) and 51.9 ± 6.0% (p = 0.011) at days 60, 90 and 120, respectively. By contrast, the reductions for the same time points for the placebo cohort, 21.9 ± 10.2% (p = 0.017), 22.2 ± 15.5% (p = 0.007) and 30.0 ± 11.8% (p = 0.012), were of lower magnitude but nonetheless statistically significant versus baseline. None of these between group differences achieved statistical significance.

### Time to complete loss of knee joint pain

During the course of this study it was noted that a number of subjects in both the placebo and the supplemented cohorts no longer reported any pain during the stepmill protocol. For the UC-II group, 5 subjects (21%) no longer reported pain by day 120, whereas only 1 subject (5%) in placebo group reported complete loss of pain (Table 5). This effect did not reach statistical significance between groups but there was an evident trend in the data towards a greater number of subjects losing pain in the UC-II cohort (p = 0.126). A binomial analysis for complete loss of pain at each visit demonstrated a statistical significance for the UC-II group by day 120 (p = 0.031). It is important to note that the complete loss of knee pain was not a random event. The pattern among the subjects indicates that loss of knee pain appeared to be a persistent phenomenon that spanned multiple visits (Table 5). A detailed review of the clinical report forms showed that none of these individuals consumed pain relief medication prior to their visits.

### Six-minute timed walk & Daily number of steps

No significant differences were observed between the study groups for the six-minute time walk or the daily number of steps taken (p > 0.05). The distance walked in six-minutes by the UC-II (range = 505 to 522 meters) and the placebo (range = 461 to 502 meters) groups were within the reference range previously reported [39] for healthy adults (399 to 778 meters, males; 310 to 664 meters, females). Similarly, the average step length calculated from Fitbit data for both study groups (0.69 to 0.71 meters) also agreed with previously published results for normal adults [40].

### KOOS knee survey & Stanford exercise scales

No significant differences were seen between the study groups for either the KOOS survey or the Stanford exercise scale (p > 0.05).

### Use of analgesics and NSAIDs

Review of the clinical report forms showed that no subject in either study cohort consumed any of the 43 prohibited medicines or supplements during the study.

### Safety assessments

A total of eight adverse events, equally dispersed between both groups, were noted (Table 6). None of the adverse events was considered to be associated with UC-II supplementation. All events resolved spontaneously without the need for further intervention. No subject withdrew from the study due to an adverse event. Finally, no differences were observed in vital signs after seventeen weeks of supplementation, and no serious adverse events were reported in this study.

**Table 6:** Summary of analysis of adverse events (AEs) in all subjects

**Study groups**	**Adverse event**	**Number of AEs**
	**(Body system)**	
UC-II	Upper respiratory infection (Pulmonary)	3
	Food poisoning (Gastrointestinal)	1
**Total number of AEs**		**4**
**Total number of subjects reporting AEs: n**		**4/27**
Placebo	Bilateral ankle edema (Musculoskeletal)	1
	Right ankle fracture (Musculoskeletal)	1
	Sinusitis (Ears/Nose/Throat)	1
	Skin infection right ankle (Dermatological)	1
**Total number of AEs**		**4**
**Total number of subjects reporting AEs: n**		**2/28**

## Discussion

In this study, the UC-II supplement, consisting of undenatured type II collagen, was investigated for its ability to improve joint function in healthy subjects who develop joint pain while undergoing strenuous exercise. The rationale behind this approach centered on the hypothesis that strenuous exercise might uncover transient joint changes due to daily physical activities that are not attributable to a diagnosable disease. In the same way that nominally elevated blood levels of lipids, glucose plus high blood pressure and obesity can be predictive of future progression to diabetes and heart disease [41], the development of joint pain upon strenuous exercise may be indicative of possible future joint problems.

At study conclusion, we found that subjects ingesting the UC-II supplement experienced a significantly greater forward ROM in their knees versus baseline and placebo as measured by knee extension goniometry. Knee extension is necessary for daily function and sport activities. Loss of knee extension has been shown to negatively impact the function of the lower extremity [42,43]. For example, loss of knee extension can cause altered gait patterns affecting ankles and the hip which could result in difficulty with running and jumping [42,43]. Studies have further shown that a permanent loss of 3-5º of extension can significantly impact patient satisfaction and the development of early arthritis [44].

By contrast, when knee flexion, another measure of knee function, was assessed via goniometry, no differences in clinical outcomes were observed between the two study cohorts. From a structure-function perspective this outcome is not surprising. During the earliest characterized phases of OA there is an apparent preferential loss of knee extension over knee flexion, and this loss has been shown to correlate with WOMAC pain scores [45,46]. In addition, MRI imaging of the early osteoarthritic knee has shown that initial changes in knee structure appear to center on articular cartilage erosions (fibrillations) about the patella and other weight bearing regions of the knee [47]. Such changes might favor a loss in knee ROM that preferentially affects extension over flexion. The pathophysiology of the early osteoarthritic knee, we believe, provides insight regarding the effect of daily physical activities on the healthy knee insofar as it helps explain the discordance in clinical outcomes between knee extension and flexion.

Both the time to onset of initial joint pain as well as time to full recovery were measured in this study. For each of these measures the clinical outcomes favored the UC-II supplemented cohort versus their baseline status. The ability of UC-II to modulate knee extension may relate to its ability to moderate knee joint pain. Crowley et al. [26] and Trentham et al. [25] demonstrated that UC-II effectively enhances joint comfort and flexibility thereby improving the quality of life (QoL) in both OA and RA subjects, respectively. This effect may be attributable to the finding that microgram quantities of undenatured type II collagen moderate CIA in both the rat and the mouse via the induction of T regulator cells [27,28,48]. The induction of these T regulators takes place within gut associated lymphatic tissues (GALT), including mesenteric lymph nodes, in response to the consumption of undenatured type II collagen [27]. Studies have shown that these regulatory cells produce IL-10 and TGF-β [30,49]. A special class of CD103^+^ dendritic cells, found almost exclusively in the GALT, facilitates this process [48,50]. Once activated, T regulator cells appear to downregulate a wide range of immunologic and proinflammatory activities resulting in the moderation of the arthritic response initiated by undenatured type II collagen [27]. The phenomenon of oral tolerance has also been demonstrated in humans, and appears to involve a similar set of T regulators [30,51-53].

The above description of how UC-II might modulate joint function is most easily understood in the context of RA given that the CIA animal model resembles this disease most closely [27,28,54]. However, the case for T regulators and immune cytokines having a moderating effect on healthy or OA knee joint function appears less apparent. This view has changed in recent years due to a growing body of evidence suggesting that both OA and normal chondrocyte biology appears to be regulated by some of the same cytokines and chemokines that regulate inflammation [5,6,55]. For example, Mannelli and coworkers [56] recently reported that feeding microgram amounts of native type II collagen (porcine) prevents monoiodoacetate-induced articular cartilage damage in this rat model of osteoarthritis, as measured by pain thresholds and by circulating levels of cross linked c-telopeptides derived from type II collagen. This finding corroborates the efficacy of undenatured type II collagen in improving joint comfort in osteoarthritic conditions [26].

In the present study, we show for the first time that UC-II can improve joint function in healthy subjects undergoing strenuous physical exercise. This observation, when considered in context with normal chondrocyte physiology, suggests that activated T regulator cells, specific for undenatured type II collagen, home to an overstressed knee joint where their release of the anti-inflammatory cytokines, IL-10 and TGF-β reverse the catabolic changes caused by strenuous exertion [13,21,57]. In addition, the IL-10 and TGF-β produced by these T regulators may tilt the T_H_ balance in the knee joint towards T_H_2 [30,58] responses which preferentially result in IL-4 production further fostering a shift in chondrocyte metabolism towards ECM replenishment.

Several additional tests were used in this study to assess overall joint function, QoL, and physical activity. The additional parameters and tests measured included a six minute timed walk plus the Stanford exercise scale and KOOS survey. With respect to the KOOS survey, both cohorts were statistically significant versus baseline for symptoms, pain, daily function, recreational activities and QoL but were not significant from each other. This is not an unexpected finding given that this study was carried out with healthy subjects who do not present with any joint issues at rest. It is only when the knee is stressed via the stepmill do subjects report any joint discomfort. Under these conditions, and as indicated above, the UC-II group appears to experience less joint discomfort and greater joint flexibility. No difference in clinical outcomes between groups was seen in the six minute timed walk, the daily distance walked, or the Stanford exercise scale questionnaire. Once again we are not surprised by these results given that these tests and questionnaires are designed and clinically validated to assess the severity of arthritic disease in unhealthy populations.

No clinical biomarkers associated with arthritic diseases were assessed in this study. Healthy subjects would not be expected to present with significant alterations in their inflammatory biomarker profile as they lack clinical disease [59]. In addition, it should be noted that the joint discomfort measured in this study is acute pain induced by a stressor rather than due to an ongoing inflammatory event. Therefore, any elevation in inflammation markers that might occur in these healthy subjects may simply be due to the physiological impact of strenuous exercise.

There are two study limitations to consider when reviewing these results. The first, time to onset of initial pain, was limited to a 10-minute interval. The current study design did not address the possibility that subjects might cease to experience pain on the stepmill. Future studies should allow for an extension of the exertion interval in order to gauge how much longer a subject can exercise before reporting pain. In this way better defined parameters can be placed upon the degree to which UC-II supplementation results in the cessation of joint pain due to strenuous exercise in healthy subjects.

The second limitation that merits consideration is the possibility that study subjects may have early signs of arthritis that do not meet the ACR criteria. This possible limitation was addressed by performing an extensive medical examination for signs and symptoms of OA and by excluding volunteers who experienced pain levels of 5 or greater within one minute of using the stepmill.

UC-II is a unique ingredient that supports healthy joints. Previous studies have focused on the efficacy of this ingredient in OA subjects. By including healthy subjects in this study, and using non-disease endpoints as a measure of efficacy, it is believed that the benefits that derive from UC-II usage now extends to include healthy individuals. Further, this ingredient appears to be safe for human consumption based on an extensive series of *in vivo* and *in vitro* toxicological studies as well as the absence of any adverse events in this and in previous human studies [24,26,60]. In conclusion, daily supplementation with 40 mg of UC-II supports joint function and flexibility in healthy subjects as demonstrated by greater knee extension and has the potential both to alleviate the joint pain that occasionally arises from strenuous exercise as well as to lengthen periods of pain free exertion.

## Abbreviations

RA: Rheumatoid arthritis; OA: Osteoarthritis; ECM: Extracellular matrix; TNF-α: Tumor necrosis factor-alpha; IL-1β: Interleukin-1 beta; IL-6: Interleukin-6; IL-4: Interleukin 4; IL-10: Interleukin-10; MMP: Matrix metalloproteinase; NF-κB: Nuclear factor-kappa-light-chain-enhancer of activated B cells; MAPK: Mitogen activated protein kinase; ERK: Extracellular receptor kinase; NO: Nitric oxide; TGF-β: Transforming growth factor-beta; CIA: Collagen induced arthritis; KOOS: Knee injury and osteoarthritis outcome score; ROM: Range of motion; MRI: Magnetic resonance imaging; GALT: Gut associated lymphatic tissue; QoL: Quality of life; MIP-1β: Macrophage inflammatory protein-1 beta; IP-10: Interferon gamma-induced protein 10; TH: T helper cell; WOMAC: Western Ontario and McMaster universities osteoarthritis index; ACR: American College of Rheumatology.

## Competing interests

Medicus Research received research grants from InterHealth Nutraceuticals, Inc., Benicia, California. Dr. Udani has provided consulting services to InterHealth Nutraceuticals, Inc. Drs. JPL, ZMS, and FCL are employees of InterHealth Nutraceuticals, Inc. Medicus Research does not endorse any brand or product nor does it have any financial interests with any supplement manufacturer or distributor.

## Authors’ contributions

JKU was the principal investigator and together with JPL, JKU, ZMS, FCL JPM, MNP and ANS contributed to the writing, data analyses and data interpretation that are a part of this manuscript. All the authors read and approved the final draft of the manuscript.
